# Hypertension Prevalence, Treatment and Control in Older Adults in a Brazilian Capital City

**DOI:** 10.5935/abc.20180274

**Published:** 2019-03

**Authors:** Ana Luiza Lima Sousa, Sandro Rodrigues Batista, Andrea Cristina Sousa, Jade Alves S. Pacheco, Priscila Valverde de Oliveira Vitorino, Valéria Pagotto

**Affiliations:** 1 Faculdade de Enfermagem, Universidade Federal de Goiás (UFG), Goiânia, GO - Brazil; 2 Faculdade de Medicina, Universidade Federal de Goiás (UFG), Goiânia, GO - Brazil; 3 Escola de Ciências Sociais e da Saúde - Pontifícia Universidade Católica de Goiás, Goiânia, GO - Brazil

**Keywords:** Hypertension/epidemiology, Hypertension/prevention and control, Prevalence, Aging, Blood Pressure, Cross-Sectional Studies

## Abstract

**Background:**

The diagnosis, treatment and control of arterial hypertension are fundamental for a reduction in cardiovascular outcomes, especially in the elderly. In Brazil, there are few studies that specifically identified these rates in the elderly population.

**Objective:**

To verify rates of prevalence, treatment and control of hypertension in elderly people living in the urban area of a Brazilian capital city.

**Methods:**

A cross-sectional, population-based, randomized, cluster-based study with 912 non-institutionalized elderly individuals (≥ 60 years), living in urban areas in the city of Goiania, Midwest Brazil. Predictor variables were: age, gender, socioeconomic and lifestyle aspects. Blood pressure measurements were performed at home; patients were considered as having arterial hypertension when SBP and/or DBP ≥ 140/90 mmHg or when using antihypertensive drugs (dependent variable). Rates of hypertension treatment and control were evaluated. Variable association analyses were performed by multivariate logistic regression and level of significance was set at 5%.

**Results:**

The prevalence of arterial hypertension was 74.9%, being higher (78.6%) in men (OR 1.4, 95% CI: 1.04-1.92); the treatment rate was 72.6%, with higher rates being observed in smokers (OR 2.06, 95% CI: 1.28-3.33). The rate of hypertension control was 50.8%,being higher in women (OR 1.57, 95% CI: 1.19-2.08).

**Conclusion:**

The prevalence rates were high. Treatment and control rates were low and associated with gender, age and lifestyle, indicating the need for early and individual interventions.

## Introduction

Despite the easy diagnosis and available treatments, arterial hypertension (AH) is still an underdiagnosed disease, with low control rates.^1^^,^^2^ Information on the prevalence, knowledge of diagnosis, treatment and control among the elderly is scarce in developing countries, even though they are acknowledged as necessary for the monitoring and development of effective strategies for AH control.^2^

In Brazil, starting from the 1970s, there was a change in the population's demographic profile, going from a mostly rural society with large families and young individuals to a mainly urban society, and with a larger proportion of elderly individuals.^3^

The prevalence of AH increases as the analyzed age group changes. In Brazil, the National Health Survey showed a 44.4% prevalence of AH in individuals aged 60 to 64 years; 52.7% from 65 to 74 years and 55.5% for those aged 75 years or older.^4^ A study carried out in Tibet identified a progressive increase in this rate, with a 19% variation in the 40-year age range and 78.1% in the 70-year age range.^5^ On the other hand, the rates of knowledge of diagnosis, treatment and control were low.^6^

It can be observed that in the Brazilian elderly population, little has been analyzed beyond the prevalence data.^7^^-^^10^ Brazilian population surveys carried out in the last 20 years, considering the adult population aged > 20 years, showed a prevalence of AH varying from 28.5% in the Southeast region^11^ to up to 53.2% in the Northern region of the country.^12^ In this latter study, the rate of knowledge of the diagnosis was 63.1% and the treatment rate was 85.4%,^12^ albeit not specifically among the elderly. No population-based studies that analyzed all these rates in the elderly population in Brazil have been identified and this lack of information has been a barrier to the development of public health policies for this population.

The aim of this study was to analyze the prevalence, treatment and control of AH and the association with life habits among the elderly, living in the urban area of a capital city in Midwest Brazil.

## Methods

This is a cross-sectional population-based study, carried out through a household survey and randomized cluster sampling, from the matrix project "*Situação de saúde da população idosa do município de Goiânia-GO*" (Health Status of the Elderly Population of the Municipality of Goiânia-GO), linked to the *Rede de Vigilância à Saúde do Idoso* (REVISI) (Health Surveillance Network of the Elderly) in the State of Goiás. The methodological and sample calculation details were described in previous publications.^13^^,^^14^

This study was developed by Universidade Federal de Goiás, the Municipal Health Secretariat of Goiânia and State Health Secretariat of the state of Goiás, through REVISI, after being approved by the Research Ethics Committee of Universidade Federal de Goiás (Protocol number 050/2009), in agreement with the Declaration of Helsinki.

An epidemiological survey was carried out, with the participation of people aged 60 and over, living in their homes and in the urban area of Goiânia. The sample was calculated considering the elderly population as 7% of the total 1,249,645 inhabitants, based on the year 2007,^15^ an estimated frequency of 30% (lowest expected frequency among the variables investigated in the matrix project), a 95% confidence interval (CI), a 5% significance level, and 5% absolute precision. To the calculated sample (n = 823), 11% were added to compensate for losses, and 934 elderly were assessed. Of the total of 934 questionnaires, 22 were excluded due to data inconsistency, and the final sample consisted of 912 elderly individuals.

The study area was defined based on census sectors (CS). The sample units were the households and the elderly in elementary observation units. Firstly, the CS were identified using the Basic Urban Digital Map of Goiânia as the basic layer. The sampling process was based on the maps of blocks and allotments of the selected regions and was carried out in multiple stages starting from the identification of CS defined by the Instituto Brasileiro de Geografia e Estatística (IBGE - Brazilian Institute of Geography and Statistics).^16^ A total of 56 CS were selected, with an estimate of reaching, on average, 17 elderly individuals in each CS.

The data were collected from residents who were at home at the time of the interviewer's visit and who accepted to participate in the study by signing the Free and Informed Consent Form. If, during data collection, two consecutive households with elderly residents were identified, the second house was excluded to minimize the conglomerate and neighborhood effect. The following inclusion criteria were considered in the study: age older than 60 years and being a resident of the household. Elderly individuals who were at the household at the time of the interview but were not residents or were unable to answer for any reason (dementia, unconsciousness) were excluded. In those cases, that household was disregarded, and the next house was considered.

The interviews were carried out by researchers properly trained to apply the study forms and also for the standardization of the procedures to be performed in data collection. The interviews were carried out from November 2009 to April 2010, considering the baseline for the *Rede de Vigilância à Saúde do Idoso* (Health Surveillance Network of the Elderly) in the capital city. Further details on the method can be verified in a previous publication.^17^

At the time of data collection, information were obtained on age, gender, socioeconomic status (level of schooling, marital status and family income), modifiable risk factors (physical activity, smoking, alcohol consumption) and information on AH treatment. Blood pressure (BP) levels were also measured. 

BP was measured using an OMRON automatic device, model HEM-705CP, following the protocol of the Brazilian Guidelines.^18^ Three measurements were performed in the same arm with the person in the sitting position, following a 3-to-5 minute interval, using the last two measurements for the calculation of the mean value, providing the difference between them was not greater than 4 mmHg. This was done to reduce data dispersion. It is worth noting that appropriate cuff sizes were used according to the arm circumference, using adequate sizes (standard, obese, pediatric) that covered two-thirds of arm extension.^18^

To identify AH prevalence, the elderly were considered hypertensive if they, during data collection, had systolic pressure values ≥ 140 mmHg or diastolic pressure ≥ 90 mmHg, or if they reported regular use of antihypertensive drugs, regardless of the BP value at the time of the interview.^18^

All patients who reported antihypertensive medication use at the time of data collection and who were able to show the prescription, or the medication boxes to be verified, were considered as undergoing treatment for AH.

The individual was considered to have controlled BP when he/she reported AH treatment and the mean BP value was lower than 140/90 mmHg.

Smoking status was classified into three groups: ex-smokers, regardless of the time since they had stopped smoking, non-smokers, for those who never smoked, and smokers. Alcohol consumption was identified according to the elderly individual's response into two groups: those who reported consuming alcohol, even occasionally, and those who reported not consuming it at all. Individuals who reported regular physical activity (three or more times a week) were classified as non-sedentary, and those who practiced physical activity less than three times a week or did not practice any physical activity were classified as sedentary.

### Statistical analysis

The quantitative variables were shown with their means and medians, standard deviations and 95%CI; the categorical variables were shown, according to their frequencies, as absolute numbers and percentages. The analysis of the normal distribution of data was performed using the Kolmogorov-Smirnov test.

The software SPSS-IBM version 23 was used to analyze the data, and the odds ratios, AH prevalence, treatment and control rates were calculated, with 95%CI. The chi-square test was used to analyze the association between AH and categorical variables, and the Mann Whitney-U test of independent samples was used to analyze the association between non-parametric, continuous quantitative variables.

Multiple logistic regression analysis was used to estimate the independent effect of variables on outcomes such as AH prevalence, treatment and disease control. The variables that showed a p value < 0.20 in the bivariate analysis were tested in the multiple logistic regression models. All statistical tests were performed considering a level of significance of 5%.

## Results

**Figure 1:**
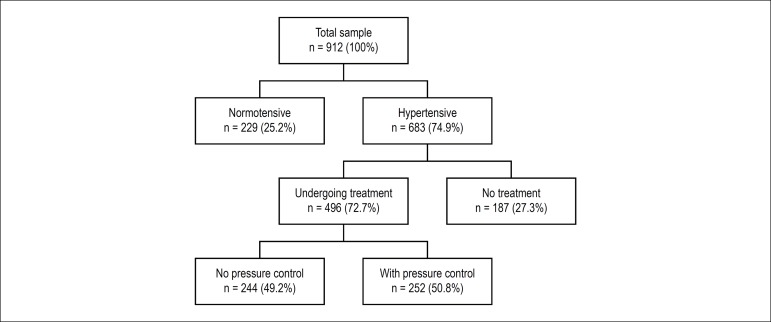
Flowchart of the assessed sample identifying normotensive and hypertensive participants; those undergoing treatment and the ones without treatment for hypertension; and with and without pressure control. Goiânia, Goiás, 2010.

Of the 912 elderly, 683 (74.9%) were hypertensive, of which 72.6% were treated for AH and, among the treated ones, 50.8% had controlled BP (Figure 1).

Of the total sample (n = 912), 62.1% were females. The mean age was 71.5 years (SD ± 8.3), and the median age was 70 years (Table 1).

**Table 1:** General characteristics of elderly individuals according to the arterial hypertension status. Goiânia, Goiás, Brazil, 2010

Variables	Hypertensive n = 683	Non-hypertensive n = 229	p	Total n = 912
	Median (Interquartile range)	Median (Interquartile range)		Median (Interquartile range)
Age (years)	70.0 (64.0 – 77.0)	70.0 (66.0 – 77.5)	0.275**	70.0 (65.0 – 77.0)
SBP (mmHg)	144.0 (126.0 – 158.0)	124.0 (114.0 – 132.5)	< 0.001**	136.5 (123.0 – 152.0)
DBP(mmHg)	81.0 (73.0 – 89.0)	73.0 (66.5 – 79.4)	< 0.001**	79.4 (70.0 – 87.0)
	**n(%)**	**n(%)**		**n(%)**
**Gender**			**0,043^†^**	
Male	272 (39.8)	74 (32.3)		346 (37.9)
Female	411 (60.2)	155 (67.7)		566 (62.1)
**Age range**			**0.906^†^**	
60 |-- 70	335 (49.0)	106 (46.3)		441 (48.4)
70 |-- 80	222 (32.5)	79 (34.5)		301 (33.0)
80 |-- 90	110 (16.1)	38 (16.6)		148 (16.2)
90 +	16 (2.3)	6 (2.6)		22 (2.4)
**Marital status**			**0.730^†^**	
Married	330 (48.3)	118 (51.5)		448 (49.1)
Single	69 (10.1)	19 (8.3)		88 (9.6)
Widowed	220 (32.2)	73 (31.9)		293 (32.1)
Divorced	61 (8.9)	19 (8.3)		80 (8.8)
**Level of schooling**			**0.831^†^**	
Illiterate	107 (15.7)	34 (14.8)		141 (15.5)
Never attended school/Can read	34 (5.0)	10 (4.4)		44 (4.8)
Complete/incomplete Elementary School	324 (47.4)	106 (46.3)		430 (47.1)
Complete/incomplete High School	144 (21.1)	50 (21.8)		194 (21.3)
Complete/incomplete College/University	67 (9.8)	24 (10.5)		91 (10.0)
Income (in MW*)			**0.173^†^**	
< 1MW	210 (30.8)	54 (23.6)		264 (29.0)
1MW |--- 2MW	284 (41.6)	110 (48.0)		394 (43.2)
2MW|--- 4MW	118 (17.3)	43 (18.8)		161 (17.7)
4MW +	70 (10.3)	22 (9.6)		92 (10.1)
**Smoker**			**0.240^†^**	
Yes	73 (10.7)	16 (7.0)		89 (9.8)
No	368 (54.1)	132 (57.9)		500 (55.1)
Ex-smoker	239 (35.1)	80 (35.1)		319 (35.1)
**Alcoholism**			**0.243^†^**	
Yes	147 (21.8)	38 (16.7)		185 (20.5)
No	459 (68.0)	162 (71.4)		621 968.8)
Ex-alcoholic	69 (10.2)	27 (11.9)		96 (10.6)
**Physical activity**			**0.374^†^**	
Yes	206 (30.2)	73 (31.9)		279 (30.6)
No	466 (68.2)	155 (67.7)		621 (68.1)
Did not answer	11 (1.6)	1 (0.4)		12 (1.3)

* MW: minimum wage (value R$ 510.00 - year 2010);

† Chi-Square Test;

** Mann Whitney U-test of independent samples.

The AH prevalence was 74.9% (n = 683), of which 431 elderly individuals were identified as having BP ≥140 and/or 90 mmHg, whereas 252 elderly individuals had BP values within the normal range but used hypertensive medication. There was a difference in prevalence between genders, being 39.8% in men and 60.2% in women.

The prevalence of isolated systolic hypertension (ISH) was 29.2% in total, with no difference between genders, being significantly higher in the age group of 70 to 80 years (112; 42.1%) when compared with the age group of 60 to 70 years (94; 35.3%), with a prevalence ratio of 1.75 (95%CI: 1.38-2.20).

Lifestyle-related variables, such as smoking status, alcohol consumption, overweight/obesity, sedentary lifestyle, level of schooling, income and marital status showed no association with AH prevalence (Table 2).

**Table 2:** Hypertension prevalence, treatment and control in elderly individuals from a Brazilian capital city. Goiânia, Goiás, 2010

Variables	Prevalence rate (n = 683) (95%CI)	Treatment rate (n = 496) (95%CI)	Control rate (n = 252) (95%CI)
Total	74.9 (69.2 – 75.9)	72.6 (69.2 – 75.9)	50.8 (44.8 – 53.6)
**Gender**			
Male	78.6 (74.1 – 82.7)	67.6 (61.9 – 73.0)	44.0 (37.0 – 51.3)
Female	72.6 (68.8 – 76.2)	75.9 (71.6 – 79.9)	54.8 (49.2 – 60.3)
p value	0.043	0.018	0.020
**Age range (years)**			
60 |-- 70	76.0 (71.8 – 79.8)	74.9 (70.1 – 79.4)	57.8 (51.6 – 63.8)
70 |-- 80	73.8 (68.6 – 78.5)	68.9 (62.6 – 74.8)	39.2 (31.7 – 47.1)
80 |-- 90	74.3 (66.8 – 80.9)	70.9 (61.9 – 78.8)	51.3 (40.2 – 62.2)
90 +	72.7 (51.7 – 88.1)	87.5 (64.5 – 97.8)	50.0 (25.1 – 74.9)
p value	0,906	0,224	0,004
**Marital status**			
Married	73.7 (69.4 – 77.6)	73.6 (68.7 – 78.2)	51.4 (45.2 – 57.7)
Single	78.4 (68.9 – 86.1)	72.5 (61.1 – 82.0)	50.0 (36.3 – 63.7)
Widowed	75.1 (69.9 – 79.8)	70.9 (64.6 – 76.6)	46.8 (39.1 – 54.6)
Divorced	76.3 (66.0 – 84.6)	72.1 (59.9 – 82.3)	63.6 (48.7 – 76.8)
p value	0.730	0.803	0.364
**Level of schooling**			
Illiterate	75.9 (68.3 – 82.4)	68.2 (59.0 – 76.5)	46.6 (35.4 – 58.0)
Never attended school/Can read	77.3 (63.2 – 87.8)	55.9 (39.1 – 71.8)	31.6 (13.9 – 54.5)
Complete/incomplete Elementary School	75.3 (71.1 – 79.2)	73.8 (68.8 – 78.3)	53.6 (47.2 – 59.8)
Complete/incomplete High School	74.2 (67.7 – 80.0)	74.3 (66.7 – 80.9)	50.5 (41.0 – 59.9)
Complete/incomplete College/University	73.6 (63.9 – 81.9)	79.1 (68.2 – 87.6)	54.7 (41.2 – 67.7)
p value	0.986	0.104	0.359
**Income (in MW)**			
< 1 SMW	79.5 (74.4 – 84.1)	74.3 (68.1 – 79.8)	55.8 (47.9 – 63.4)
1MW |--- 2MW	72.1 (67.5 – 76.3)	72.9 (67.5 – 77.8)	44.9 (38.2 – 51.8)
2 MW|--- 4MW	73.3 (66.1 – 79.7)	70.3 (61.6 – 78.1)	51.8 (41.1 – 62.4)
4MW +	76.1 (66.6 – 83.6)	71.4 (60.1 – 81.1)	58.0 (44.1 – 71.0)
p value	0.173	0.883	0.141
**Smoker**			
Yes	82.0 (73.0 – 89.0)	84.9 (75.3 – 91.8)	46.8 (34.6 – 59.2)
No	73.6 (69.6 – 77.3)	72.8 (68.11 – 77.2)	51.5 (45.5 – 57.4)
Ex-smoker	74.9 (69.9 – 79.4)	68.6 (62.5 – 74.3)	51.8 (44.2 – 59.4)
p value	0.240	0.024	0.773
**Alcoholism**			
Yes	79.5 (73.2 – 84.8)	75.5 (68.1 – 81.9)	38.7 (30.2 – 48.0)
No	73.9 (70.4 – 77.2)	71.2 (67.0 – 75.2)	53.2 (47.8 – 58.6)
Ex-alcoholic	71.9 (62.3 – 80.2)	73.9 (62.6 – 83.2)	58.8 (45.0 – 71.7)
p value	0.243	0.577	0.014
**Physical activity**			
Yes	73.8 (68.4 – 78.7)	75.2 (69.0 – 80.8)	51.0 (43.1 – 58.8)
No	75.0 (71.5 – 78.3)	71.2 (67.0 – 75.2)	50.6 (45.2 – 56.0)
p value	0.374	0.285	0.940

MW: minimum wage (value R $ 510.00 - year 2010).

Of the 431 individuals who were identified as having altered BP levels, 187 (43.4%) were unaware of the probable AH diagnosis and were not being treated for the disease. Of the 683 patients considered hypertensive, 496 (72.6%) reported regular use of antihypertensive medication, with lower rates (67.6%) being observed in men when compared to women (75.9%) (Table 2).

Among those who received treatment for the disease, 252 (50.8%) showed BP control (SBP/DBP < 140/90mmHg), also with a difference between genders; the control rates were higher among those aged 60 to 70 years (Table 2).

As for alcohol consumption, there was an association with the control rate, with lower control rates being observed among those who consumed alcohol (Table 2).

The multiple logistic regression analysis showed there was a significant association between the prevalence rate and the male gender, with a higher probability of AH (OR = 1.39, 95%CI 1.04-1.92). Current smoker was associeted with the treatment rate (OR = 2.06, 95%CI: 1.28-3.33). Female gender (OR = 1.57, 1.19-2.08) and alcohol consumption (OR = 1.41, 95%CI 1.00-1.99) were associated with the control rate (Table 3).

**Table 3:** Multivariate logistic regression analysis of the factors associated with the analyzed rates

Variables	Adjusted Odds Ratio (95%CI)	Wald test	p value
**Prevalence of arterial hypertension**			
Age (years)	1.01 (0.99 – 1.02)	0.25	0.614
**Gender**			
Female	1		
Male	1.39 (1.04 – 1.92)	4.16	0.041
**Income (in MW)**			
< 1MW	1		
1MW |--- 2MW	0.79 (0.45 – 1.4)	0.64	0.423
2 MW|--- 4MW	1.17 (0.69 -- 2.00)	0.34	0.559
4MW +	1.12 (1.00 – 1.02)	0.25	0.614
**Treatment rate**			
Age (years)	1,00 (0,99 – 1,02)	0,11	0,740
**Gender**			
Female	1		
Male	1.12 (0.85 – 1.47)	0.66	0.417
**Smoker**			
Non/ex-smoker	1		
Yes	2.06 (1.28 – 3.33)	3.22	0.003
**Control rate**			
Age (years)	0.99 (0.97 – 1.00)	2.30	0.130
**Gender**			
Male	1		
Female	1.57 (1.19 – 2.08)	9.93	0.002
**Alcohol consumption**			
Yes	1		
No	1.41 (1.00 – 1.99)	3.88	0.049

Other studies carried out with the elderly found a greater proportion of women undergoing treatment.

## Discussion

The present study analyzed the prevalence, treatment and control rates of AH in a representative sample of the urban elderly population in the city of Goiânia, Brazil. The prevalence of AH was 74.9%, higher than observed in the country's adult population shown by other studies carried out in different regions.^8^^,^^11^^,^^12^^,^^19^^-^^21^ The prevalence of AH in individuals aged between 50 and 70 years is approximately 6 to 8-fold higher than that in young adults, aged between 18 and 29 years,^19^^-^^22^ consistent with 16 studies carried out in the country between 1989 and 2007, which reported prevalence rates of AH higher than 60% in the elderly population.^10^

Similar prevalence rates were also shown in a study carried out in Poland, with lower values being observed in men (69.9%; 95%CI: 65.2-74.2) than in women (80.2%, 95%CI: 75.7-84.1), at the age range of 80 years old or older. The prevalence for Polish individuals older than 65 years (BP140/90 mmHg) was 78.2% (95%CI: 76.44-79.8) in women and 70.1% (95%CI 68.2-71.8) in men,^22^ in opposition to our study, where women showed lower rates. The difference in AH prevalence between genders has been previously described in several studies carried out in different countries, as well as the association with age.^23^^-^^26^ Until the age of 60 years, the proportion of hypertensive women is lower because they rely on the hormonal protection of estrogens, whereas it is predicted that these rates will be equal between men and women after the latter go through menopause.^24^

Similarly, our study showed that ISH had higher prevalence rates among those aged 70 years and older, with no difference between genders, which contrasts with the Polish study that found higher ISH rates in men older than 85 years.^22^

The treatment rate found in our study was higher among women and showed no association with the different age groups. Other studies carried out with the elderly found a greater proportion of women undergoing treatment. Treatment is related to the access to health services, as well as the level of knowledge of AH diagnosis and the prevalence.^28^^,^^29^ In our country, the identification of these rates, whether among the general population or in specific age groups, comes from population-based surveys or specific studies under certain conditions, such as implemented programs.^30^ The difficulty of having access to and receiving care at health services do not allow the opportunity for diagnosis and treatment. This is even more serious when it is related to elderly individuals with AH who are unaware of their diagnosis.

The blood pressure control rates found in our study among those who received treatment for AH were low and significantly lower in men. According to data from the PURE study, which analyzed data from 17 countries representing five continents, AH treatment and control rates in South American countries were lower than those found in our study, even when considering the specific age range of the elderly.^2^

Despite the efforts of health professionals at all levels, blood pressure control rates worldwide are only reasonable. Canada has a rate of 64.6%,^31^ Switzerland has 59.4%,^32^ the United States has 57%^33^ and England, 37%.^34^ In Brazil, these rates vary between 22.5% in the North region^11^ and 24.2% in the Midwest.^20^

Ignoring a high BP rate is a risk to one's cardiovascular and renal health, as it increases the chances of life-threatening complications, and the higher the BP, the greater the risk of consequences for the heart and blood vessels in the major organs, such as the brain and kidneys, regardless of the age range.^29^

## Conclusions

The prevalence and treatment rates of AH found in this study's population were high, 74.9% and 72.6%, respectively. However, only 50.8% of the individuals achieved their blood pressure control targets. Women showed higher rates of treatment and control when compared to men.
